# Material stock and environmental burdens of coastal bridge infrastructure in China: A bottom-up life cycle perspective

**DOI:** 10.1371/journal.pone.0339684

**Published:** 2026-03-18

**Authors:** Huanshan Ning, Zhen Guo, Haibo Feng, Peidong Zhang, Jiawei Shen, Zhiwei Zhang

**Affiliations:** 1 College of Geodesy and Geomatics, Shandong University of Science and Technology, Qingdao, PR China; 2 Research Center for Islands & Coastal Zone, First Institute of Oceanography, Ministry of Natural Resources, Qingdao, PR China; 3 Department of Wood Science, the University of British Columbia, Vancouver, BC, Canada; 4 College of Environment and Safety Engineering, Qingdao University of Science and Technology, Qingdao, China; Koneru Lakshmaiah Education Foundation / Indian and Xidian University, INDIA

## Abstract

China’s rapid expansion of maritime infrastructure has positioned coastal bridges as critical components of national economic and transport strategies. However, their material stock scale, material intensity and long-term environmental impacts remain underexamined. This study presents the first integrated assessment of material stock (MS) and lifecycle environmental burdens associated with China’s coastal bridges, using a bottom-up material flow analysis and ReCiPe 2016 midpoint life cycle assessment (LCA). Results based on 510 coastal bridges show a total in-use material stock of 369.32 million tons, with cable-stayed bridges accounting for 63.9%. Suspension bridges exhibit the highest material intensity (18.78 t/km), primarily due to anchorage systems requiring 12.4 times more crushed stone and 4.7 times more concrete than cable-stayed designs. Life cycle assessment reveals that raw material production contributes 66.6% of total environmental impacts, with steel production alone accounting for 60.9% of global warming potential (GWP) and over 90% of mineral scarcity and human carcinogenicity. Although steel comprises only 13.9% of MS, its unit mass impacts are up to 380 times greater than those of sand and gravel. In addition, hidden flows associated with structural overdesign, anticorrosive coatings, and deferred maintenance shift substantial burdens to upstream phases, contributing 27.8% of GWP and 49.9% of marine ecotoxicity. Mitigation strategies include dynamic LCA frameworks, circular material flows, modular low-carbon construction, bio-based coatings, and alignment with global carbon governance. These findings provide practical guidance for advancing low-carbon, climate-resilient coastal infrastructure in China.

## 1. Introduction

Material resources, a key driver of economic growth and social development, have seen a tremendous increase in global consumption. According to 2024 UNEP IRP data, resource consumption surged from 30 billion tons in 1970–106 billion tons, with extraction projected to rise by 60% to 170 billion tons by 2060 [1]. Throughout their lifecycle, from exploration to utilization, these resources have led to significant challenges, including energy shortages, climate change, environmental degradation, and habitat loss. Sustainable management of material resources has become a crucial topic for balancing socio-economic development with responsible material use. It also constitutes a key pillar of the United Nations’ 2030 Sustainable Development Goals (SDGs) [2–4].

A Material Stock (MS) study is a research approach that aims to quantify and analyze the total volume of materials embedded in products, infrastructure, and buildings within a given system. By examining how these materials are accumulated and utilized in society or sectors, researchers and policymakers can develop more effective strategies to enhance resource efficiency [5,6], minimize waste, and promote long-term sustainability.

For 40 years since reform and opening up, China has adhered to the “infrastructure first” policy and achieved remarkable accomplishments in infrastructure construction, rapidly becoming the world’s largest infrastructure market. Notably, China’s densely populated and economically vibrant coastal zones have witnessed astonishing growth in artificial infrastructure stock in recent years, driven by coastal urbanization and the implementation of the national marine economic strategy. The length of artificial shoreline along China’s mainland coastline, totaling approximately 18,000 km, has sharply increased from 3,000 km in 1940–13,000 km in 2020 [7–9]. The scale of marine infrastructure facilities, including ports and terminals, artificial embankments, cross-sea bridges and tunnels, and offshore wind farms, has also expanded rapidly. These developments have become a key driver of economic growth and a vital component of the artificial structure stock in coastal zones [10,11].

Therefore, conducting in-use material flow analysis of maritime infrastructure systems, while clarifying their lifecycle impacts and identifying the main sources and stages of carbon emissions, is crucial for understanding material occupation and energy input under the influence of intensive human activities in China’s coastal areas.

## 2. Literature review

For the past three decades, scholars have systematically examined MS within the framework of urban metabolism (UM), positioning it as a critical area of inquiry in socio-economic system analysis. These studies emphasized the substantial influx of material resources from the geosphere into the anthroposphere and their continuous accumulation within urban systems, resulting in the formation of long-life-cycle buildings [12–15], infrastructure [16–19], and durable consumer goods [20–23]. Simultaneously, there is growing awareness that large-scale stock accumulation not only demands vast material and energy inputs but also generates significant waste flows as materials transition from the usage phase to renewal, maintenance, and disposal. This shift imposes considerable ecological risks and environmental pressures on surrounding ecosystems.

Studies indicate that the global building and infrastructure sector accounts for over 40% of total resource consumption [24–34], leading to increasing attention on infrastructure in MS research in recent years. For example, changes in urban infrastructure stock across Beijing, Tianjin, and Shanghai (1978–2013) have been examined using Material Flow Analysis (MFA), Remote Sensing (RS), and Geographic Information Systems (GIS) [35], while bottom-up modeling has been applied to assess in-use stock and composition, particularly in Beijing’s urban road system [36]. A bibliometric analysis of 249 studies (1985–2018) has compared top-down and bottom-up approaches in built environment research [37]. Similarly, bottom-up modeling has been used to evaluate material flows and embodied emissions in road and rail infrastructure, including tunnels and bridges, across 180 countries in 2021 [38]. To address data availability challenges, Google Earth has been leveraged to improve MFA data accuracy, particularly for analyzing French railway infrastructure [18]. Additionally, material intensities for various building types have been derived from material composition data, building structure codes, and GIS/RS data from municipal authorities. The calculation of MS primarily relies on two major architectural frameworks: the top-down macro analysis and the bottom-up micro construction [39–41]. A growing trend in MS studies is integrating MS data with multi-source inputs to enhance bottom-up methodologies [17,18,42–44], aiming to address challenges such as inaccuracies in non-physical stock assessments and the low precision of macro-level statistical data.

Coastal bridges constitute important transportation infrastructure linking land and sea, characterized by high material demand, construction energy consumption, long service life, and significant environmental impact. Assessing bridge MS offers insight into recovery potential, can optimize future construction and maintenance, improve resource efficiency, quantify life-cycle carbon emissions and ecological impacts, thus supporting sustainable infrastructure development. Recent research programs have begun addressing these challenges: Lange et al. [45] evaluated urban bridge material inventory by compiling data on number, construction date, and location of bridges in North Rhine Westphalia, predicting substantial material flows over the next 20 years. Lantsot et al. [46]. conducted collapse tests on reinforced concrete slab bridges in the Netherlands to understand dynamic modeling of MS degradation. Xia et al. [47]. conducted a carbon emission life cycle assessment on Maogang bridge in Shanghai, predicting carbon emission trends through 2050. However, few existing studies integrate technical data with socio-economic cycle indicators, emphasizing the need for a spatial solution and comprehensive life cycle analysis framework. Bridge data, including material composition, is often difficult to obtain due to contractor proprietary information and differing design standards, highlighting the interdisciplinary nature of such research. Although crucial, no relevant research reports exist to date.

This study focuses on coastal bridge systems along China’s coastline, classifies the stock system based on bridge structures, and develops a bottom-up stock calculation model to estimate in-use MS of coastal bridge system and further analyze lifecycle carbon emission characteristics. The study aims to provide methodological and case support for sustainable maritime infrastructure resource management and corresponding emission decision-making guidance to achieve China’s “carbon peak and neutrality” goals.

## 3. Materials and methods

### 3.1. MS calculation model for coastal bridges

**Fig 1:**
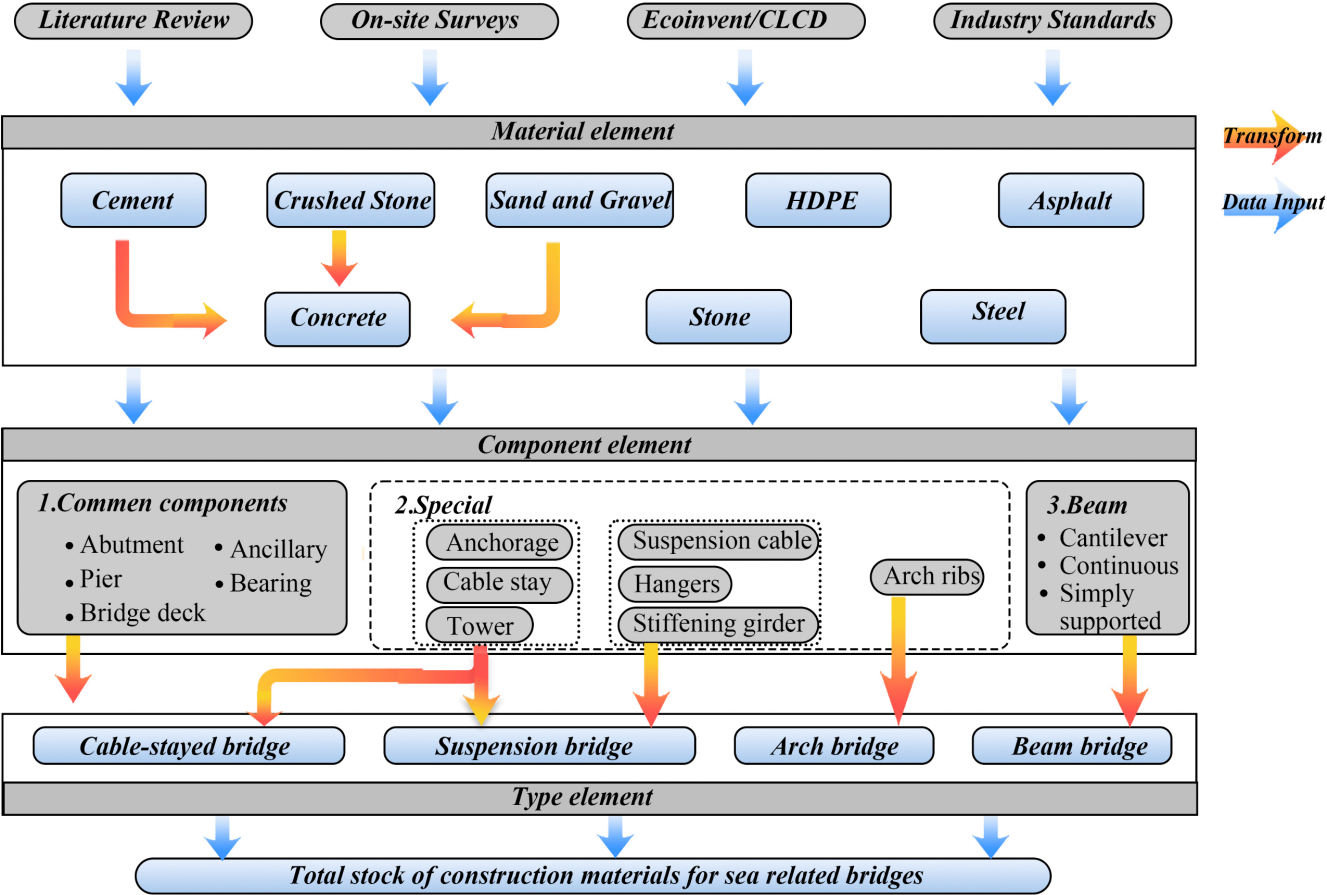
Classification system for MS of coastal bridges based on a bottom-up framework.

Compared to the top-down approach, which provides a broad estimation of MS, the bottom-up framework emphasizes collecting detailed data from specific case studies to analyze the internal material composition and address the “black-box” nature of MS systems. In this study, the bottom-up approach was used to systematically classify the MS of sea-related bridges into four distinct categories based on their functional and structural characteristics: cable-stayed bridges, suspension bridges, arch bridges, and beam bridges (Fig 1). The accounting covers major construction materials, including cement, concrete, crushed stone, sand and gravel, high-density polyethylene (HDPE), asphalt, stone, and steel.

Caption credit: Each type of bridge is further deconstructed according to its constituent components.

The Structural Decomposition Material Intensity (SDMI) method is used to divide the common structures of a specific type of sea-related bridge into distinct structural units. The material intensity contained within each structural unit is quantified through a comprehensive analysis of structural design drawings, construction records, actual measurements, and expert consultation. The MS for each structural unit is calculated, enabling the determination of the total MS for that specific bridge type. The calculation model is formulated as follows:


$$S_{t}=\sum_{i}M_{i,t}$$ (1)

$$M_{i,t}=\sum_{i}{[N}_{i}(\rho_{i,t}\times V_{i,t})]$$ (2)

where:

$S_{t}$ represents the total MS of type *t* for the studied object *i*, measured in ten thousand tons;

$M_{i,t}$ represents the mass of MS *t* used in structural unit type *i*, measured in ten thousand tons;

$N_{i}$ represents the scale or quantity of structural unit type *i*;

$\rho_{i,t}$ represents the density of material *t* within structural unit type *i*;

$V_{i,t}$ represents the volume of material *t* used in structural unit type *i*, measured in cubic meters ($m^{\text{3}}$).

In applying the bottom-up material flow estimation model, this study accounted for the structural characteristics of each bridge type and relevant construction environmental conditions affecting material use. Where precise data were lacking, average values from comparable projects and expert input were used to ensure realistic estimates. Table 1 highlights the key materials for each bridge type, reflecting variations driven by structural design, geographic context, construction methods, and material availability. These factors were integrated into the estimation process to enhance the accuracy and reliability of results.

**Table 1:** Material consumption focus for different bridge types in coastal infrastructure projects.

Bridge Type	Key Structural Features	Material Consumption Focus (by Material Type)	Construction Environment Requirements
Cable-Stayed Bridge	Main cables (under tension); Stay cables; Towers; Deck.	Steel: Used for main cables, stay cables, and towers; Concrete: Used for towers, deck, and foundation; Cement: Used for concrete preparation; Crushed Stone & Gravel: Used for foundation support structures; Asphalt: Used for deck surfacing.	Requires large foundation support, suitable for spanning large rivers or valleys
Suspension Bridge	Main cables (under extreme tension); Anchor blocks; Towers; Suspenders.	Steel: Used for main cables, suspenders, and towers; Concrete: Used for anchor blocks and tower foundations; Cement: Used for concrete preparation; Crushed Stone & Gravel: Used for filling anchor blocks and foundation structures; Asphalt: Used for deck surfacing.	High geological stability required for anchor blocks and tower foundations
Arch Bridge	Arch structure; Compressive load; Deck; Upports.	Concrete: Used for arch structure, supports, and deck; Cement: Used for concrete preparation; Crushed Stone & Gravel: Used for foundation support; Stone: Used for decorative arch rings or surface; Steel: Used for structural reinforcement.	Suitable for mountainous or canyon regions with stable geological conditions
Beam Bridge	Beam structure; Supports; Deck.	Steel: Used for beam structure and supports; Concrete: Used for foundation and deck; Cement: Used for concrete preparation; Crushed Stone & Gravel: Used for foundation support and bedding; Asphalt: Used for deck surfacing.	Flexible construction environment, adaptable to various terrain conditions

### 3.2. Life cycle assessment methods for coastal bridges

Building on the LCA framework outlined in ISO 14040/14044 and the environmental profiling methodology introduced by the BRE Group [48], this study defines system boundaries and functional units for LCA. Given that most sea-related bridges are designed for long service lives and the limited availability of reliable end-of-life (EoL) recycling data, the study adopts a “cradle-to-gate” approach, excluding the demolition and recycling stages. It should be noted that this boundary may slightly underestimate the extent to which future material recovery could offset upstream impacts. Therefore, a scenario analysis of end-of-life material recycling was conducted to assess its potential to mitigate upstream environmental impacts and support circular economy strategies.

The Life Cycle Inventory (LCI) was developed by integrating MS data, on-site surveys, industry standards, literature reviews, the China Products Carbon Footprint Factors Database (CPCD), and the Ecoinvent database. Environmental impact assessment was conducted using the ReCiPe 2016 methodology, which quantifies impacts by calculating characterization factors—indicators representing the environmental burden per unit of emissions or resource extraction [49,50]. Characterization factor derivation follows two complementary approaches: midpoint [51,52] and endpoint methods [53,54]. While endpoint methods closely align with environmental flows, midpoint methods exhibit lower uncertainty and are better suited for evaluating specific engineering systems [55].

To accurately assess the lifecycle impacts of cross-sea bridges from a “cradle-to-use” perspective—excluding end-of-life stages due to their long service lifetimes—six critical midpoint indicators were selected based on China’s environmental priorities and the specific characteristics of bridge engineering (Table 2).

**Table 2:** Environmental sustainability impact assessment indicators classification.

Index	Unit	Environmental Focus	Purpose
Global Warming Potential (GWP)	kg CO_2_ eq	Climate change (greenhouse effect)	Quantifying carbon emissions associated with climate change
Human Carcinogenic toxic	kg 1,4-DCB	Air pollution and public health	Assess the carcinogenic risk of construction emissions to health
Mineral Resource Scarcity	kg Cu eq	Depletion of non renewable resources	Solve the problem of excessive exploitation of sand and gravel in coastal areas
Fossil Resource Scarcity	kg oil eq	Energy security and Sustainability	Assess dependence on non renewable energy sources
Freshwater Ecotoxicity	kg 1,4-DCB	Pollution of freshwater ecosystem	Toxic effects of capturing construction runoff on water ecology
Marine Ecotoxicity	kg 1,4-DCB	Marine ecological destruction	Monitoring the long-term marine effects of anticorrosive coatings and sediments

This selection balances scientific rigor and policy relevance by minimizing uncertainties in characterization modeling and ensuring applicability to regional environmental challenges. Consequently, the adopted indicator set supports a robust and context-specific evaluation of coastal bridge environmental performance, providing actionable insights for sustainable infrastructure development.

### 3.3. Study area and data collection

**Fig 2:**
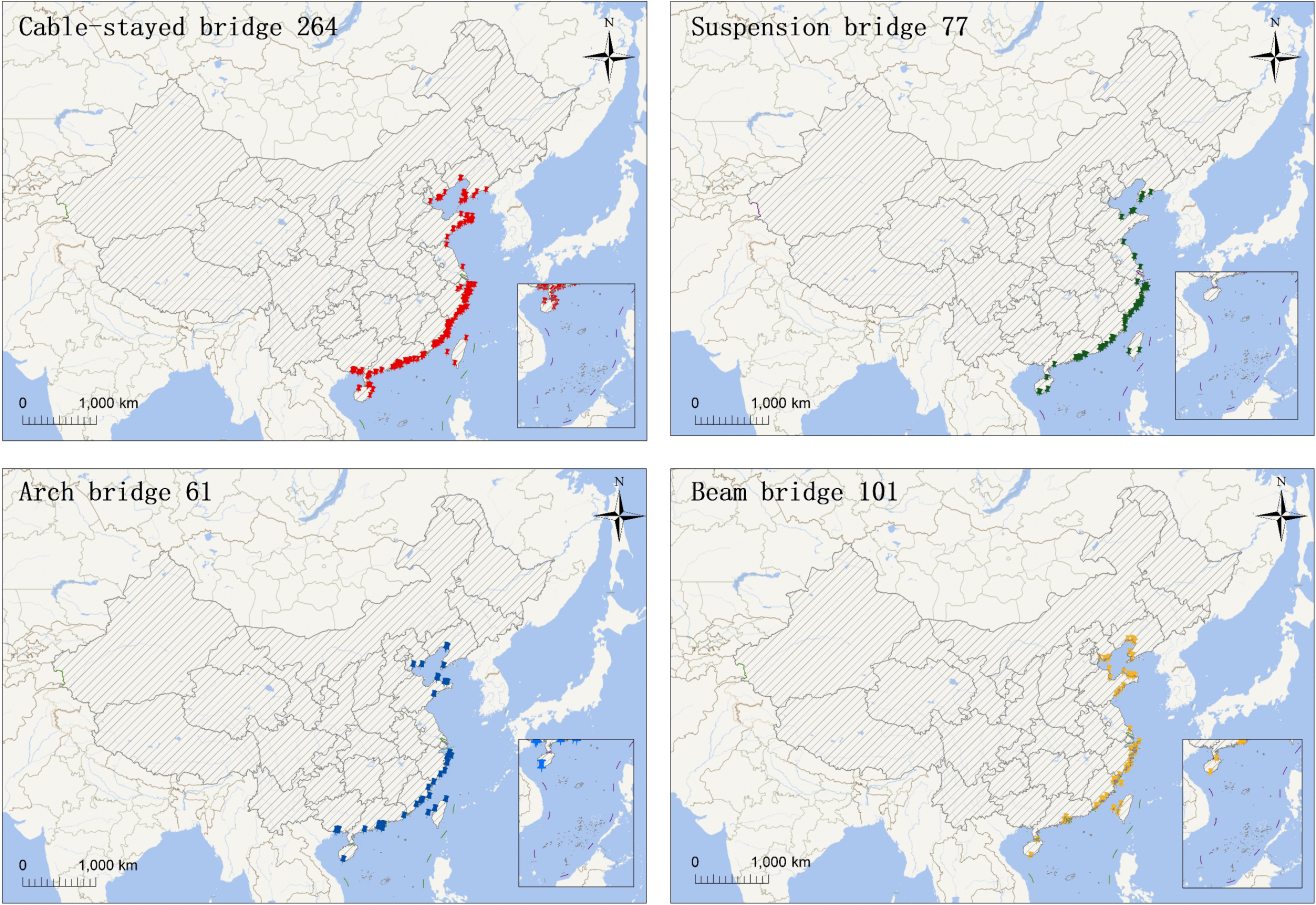
Geographical distribution of Sea-Related bridges in China by structural type. Caption credit: The four submaps illustrate the spatial distribution of cable-stayed, suspension, arch, and beam bridges along the Chinese coastline, highlighting their geographic density and typological variation.(The base map and administrative boundaries were obtained from the National Platform for Common Geospatial Information Services (Tianditu, https://www.tianditu.gov.cn/; map review No. GS(2024)0650).).

**Fig 3:**
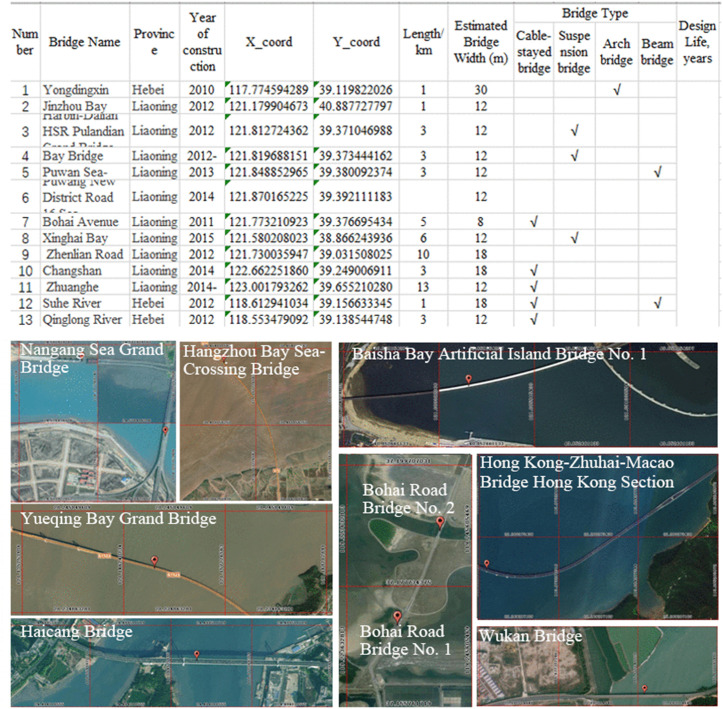
Basic information and spatial distribution of Sea-Crossing bridges in China. (The basemap was redrawn by the research team based on UAV photogrammetric data acquired between April 2019 and October 2020 using the DJI Phantom 4 RTK platform. The average flight altitude was approximately 120 m, with a ground sampling distance of 0.03–0.05 **m.** All imagery was orthorectified and vectorized. The spatial locations and basic attributes of all identified bridges are provided in the Excel file within the S1 File.).

This study focuses on evaluating material sustainability (MS) and conducting a life cycle assessment (LCA) of coastal bridge systems across China’s coastal zones. To ensure spatial accuracy and data representativeness, a nationwide spatial vector database of coastal bridges was established by integrating multi-source geospatial information. The dataset was developed using high-resolution satellite imagery (2019–2020) from the Jilin-1 satellite constellation (https://www.jl1mall.com/), complemented by UAV photogrammetric data collected in April 2019 and October 2020, and further supplemented with open-source basemap data. Coastal bridges were identified and classified through an object-based image analysis (OBIA) approach combined with machine learning algorithms, followed by manual validation to ensure accuracy. In total, 510 bridges were identified (Figs 2 and 3), distributed across 11 mainland coastal provinces and three special administrative regions (Hong Kong, Macao, and Taiwan), covering 14 coastal administrative units. The dataset includes key attributes such as bridge coordinates, structural type, length, width, and year of construction. These bridges encompass four major structural categories—cable-stayed, suspension, arch, and beam bridges. Overall, the dataset exhibits comprehensive spatial coverage, structural diversity, and temporal representativeness, providing a robust foundation for the nationwide estimation of material stocks and life cycle environmental impacts of coastal bridge infrastructure in China.

Data related to bridge design, construction practices, and carbon emissions were sourced from multiple authoritative sources, including official statistics from the National Bureau of Statistics, IPCC public datasets, Chinese engineering and data platforms, official bridge project websites, the China Products Carbon Footprint Factors Database (CPCD), and peer-reviewed academic literature. National and international technical standards and building codes were referenced to ensure methodological consistency and scientific rigor. For bridges lacking detailed engineering documentation, supplementary parameters were obtained from the Ecoinvent v3.9 database and relevant publications. The emission factors used in the life cycle inventory (LCI) are summarized in Table 3.

**Table 3:** Life cycle greenhouse gas emission factors for coastal bridge construction and operation.

Emission Source	Item	Emission Factor (kg CO₂/unit)	Notes/ Source
Fossil Energy	Raw Coal	1.9027 kg CO_2_/kg	Based on GB/T 2589−2020 General rules for calculation of comprehensive energy consumption [56]. and Technical Guidelines [57] CO₂ emission coefficient = Average Lower Calorific Value × Carbon Content per Unit Calorific Value × Carbon Oxidation Rate × (44/12) [58].
	Coke	2.8567 kg CO_2_/kg	Same calculation methodology as raw coal.
	Crude Oil	3.0240 kg CO_2_/kg	Derived from national fuel life cycle emission factors.
	Fuel Oil	3.1744 kg CO_2_/kg	Based on fuel combustion emission factors.
	Gasoline	2.9279 kg CO_2_/kg	Calculated using life cycle emission intensity of gasoline.
	Kerosene	3.0372 kg CO_2_/kg	Emission factor based on standard aviation and industrial kerosene combustion.
	Diesel	3.0998 kg CO_2_/kg	Derived from national emission inventory methodologies.
	Natural Gas	1.7928 kg CO_2_/kg	Based on the molar CO₂ emission ratio of methane combustion [59].
Anthropogenic Emissions	—	—	Total per capita carbon emissions: 0.8 t/year. Based on national carbon footprint models.
Electricity	—	0.5568 kg CO_2_/kWh	Based on the latest national electricity CO₂ emission factor released by the Ministry of Ecology [60] and Environment [58,59].
Building Materials	Concrete	385 kg CO_2_/t	Carbon footprint assessments account for emissions from production, recycling, and disposal [58]. China Products Carbon Footprint Factors Database (CPCD) (https://lca.cityghg.com/)
	Cement	735 kg CO_2_/t	CPCD (https://lca.cityghg.com/)
	Steel	2,340 kg CO_2_/t	CPCD (https://lca.cityghg.com/)
	Crushed Stone	2.18 kg CO_2_/t	CPCD (https://lca.cityghg.com/)
Construction Machinery	Heavy-Duty Trucks	2.376 kg CO_2_/km	CPCD (https://lca.cityghg.com/)

It should be noted that due to limitations in remote sensing resolution, data accessibility, and model recognition accuracy, some small or low-material-intensity bridges in rural areas (e.g., simply supported beam or stone arch bridges) may not have been fully captured. These structures are typically less than 100 m in length and mainly serve local transport. Their average material use per bridge is estimated to be only about 1–3% of that of large coastal bridges, resulting in minimal life cycle environmental impacts. Therefore, their omission introduces negligible bias to the national-scale assessment. Additionally, certain early-built bridges lack detailed records of structural and material properties, leading to uncertainties in parameters such as concrete strength and reinforcement ratio. To reduce such effects, this study applied parameter validation against historical and current design standards, ensuring the consistency and robustness of the estimates.

Overall, the constructed national coastal bridge database demonstrates comprehensive spatial coverage, structural diversity, and temporal representativeness. Despite minor uncertainties, it provides a solid empirical basis for nationwide assessment of material stocks and life cycle environmental impacts of China’s coastal bridge infrastructure.

## 4. Results

### 4.1. Material stock composition of coastal bridges

Building on the bottom-up MS framework and incorporating relevant data for China’s coastal bridges (Section 3.3), this study calculates the total in-use MS of 510 coastal bridges in China (Table 4).

**Table 4:** MS calculation results for the 510 coastal bridges along the coast of China (Unit: Million tons).

Materials	M_cable-stayed_	M_suspension_	M_arch_	M_beam_	Total amount
Cement	35.75	11.85	2.25	2.99	52.82
Sand and Gravel	74.67	20.31	4.69	5.74	105.41
Crushed Stone	16.27	27.08	1.19	1.36	45.90
Asphalt	2.87	0.77	0.17	0.20	4.01
Steel	40.18	4.86	2.94	3.36	51.34
Stone	8.80	0.80	0.57	0.73	10.90
HDPE	3.83	0.01	0.23	0.27	4.34
Concrete	53.60	33.85	3.02	4.14	94.61
Total	235.96	99.53	15.06	18.79	369.32

**Fig 4:**
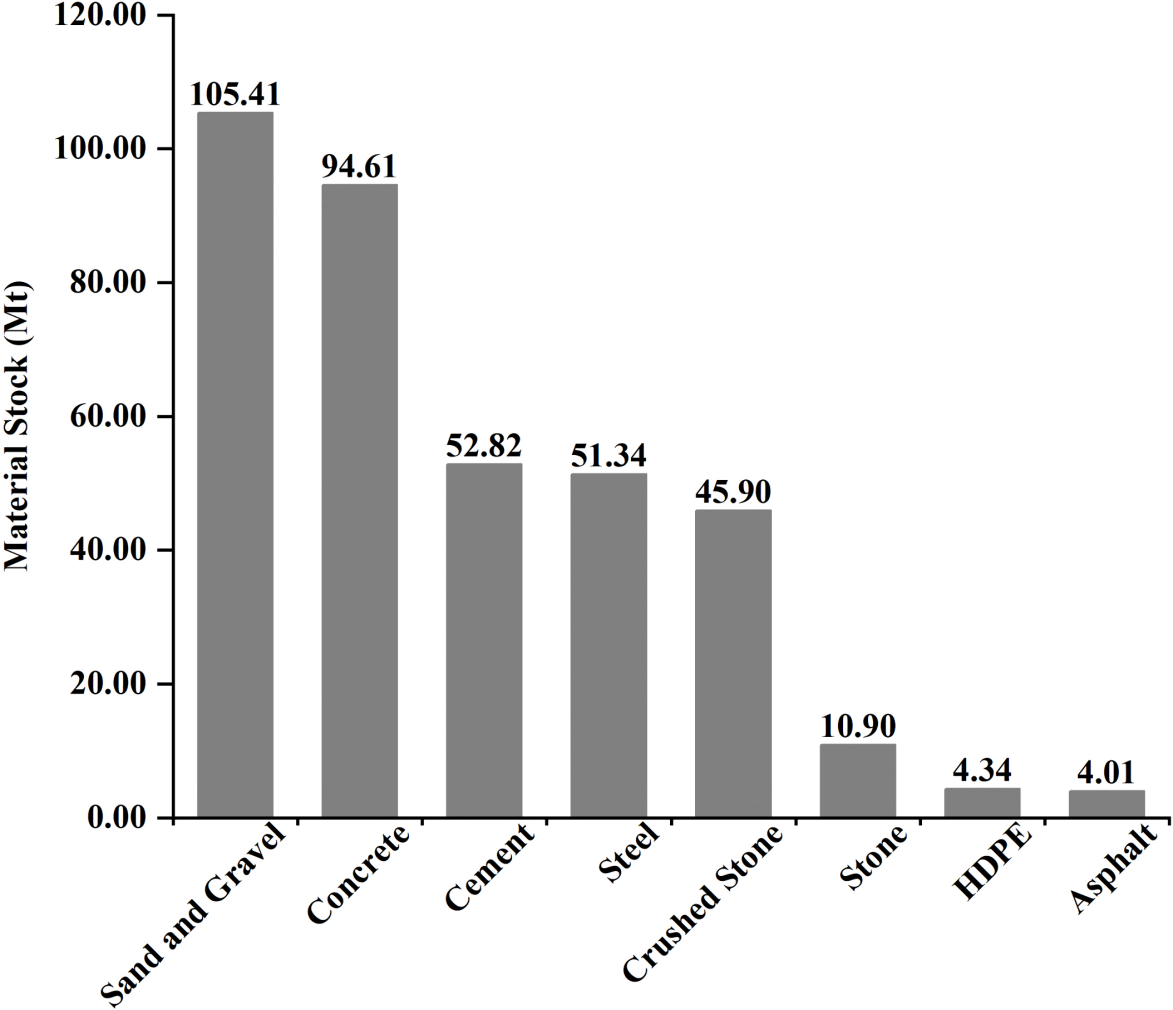
Material composition of the total MS in China’s coastal bridge system.

The calculation results show that the MS of China’s coastal bridge system is approximately 369.32 million tons (Fig 4). Among them, sand and gravel are the most used building materials, with 105.41 million tons, accounting for 28.54% of the total, mainly used as concrete and mortar aggregate to fill bridge piers, support the bridge deck, and pave roads, ensuring the stability and durability of the bridge under conditions such as seawater erosion and climate change. Cement, steel, and concrete are also indispensable building materials, accounting for 14.30%, 13.90%, and 25.62% of the total, respectively. Concrete, with a stock of 94.61 million tons, ranks as the second largest material in the total MS system, primarily used as the main material for the bearing and foundation structure of sea crossing bridges. The addition of steel reinforcement endows the concrete structure with excellent tensile and bending resistance, significantly enhancing its load-bearing potential and seismic performance. Materials with lower stocks are asphalt and HDPE, with 4.01 million tons and 4.34 million tons, respectively.

**Fig 5:**
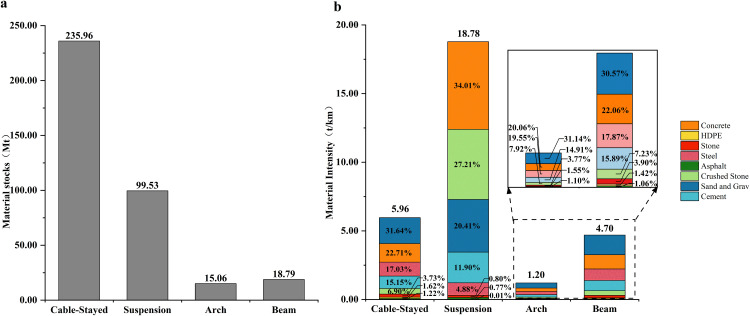
Inventory distribution and material intensity characteristics of coastal bridges(/km). **(a)** Total stock distribution by bridge type; **(b)** Material stock intensity per kilometer (breakdown by bridge type and material category).

The MS proportion (Fig 5a) reveals an overwhelming dominance of cable-stayed bridges, constituting 63.9% of the total stock, a consequence of their extensive deployment in China’s coastal megaprojects. Suspension bridges, though fewer in number (77 bridges), exhibit remarkable per-unit material intensity (18.78 t/km), as detailed in Fig 5b. This intensity is driven by anchorage and cable demands, with crushed stone (5.11 t/km, 27.21%) and concrete (6.39 t/km, 34.01%) stocks exceeding those of cable-stayed bridges by 12.4× and 4.7 × , respectively. These results emphasize the structural and material trade-offs inherent in cross-sea bridge design, advocating for span-specific optimization frameworks to reconcile engineering feasibility with sustainability goals.

### 4.2. Life cycle inventory analysis and environmental impact characterization

#### 4.2.1. Objective and scope definition.

Considering data availability and the need to maintain consistency within the defined life cycle assessment (LCA) system boundaries, this study adopts bridge length (t/km) as the functional unit for coastal bridge construction activities. A dimensional normalization approach is applied to reduce, to some extent, the parameter sensitivity caused by structural scale differences and to improve data comparability among bridge samples [61]. However, using bridge length alone as the functional unit may still underestimate the potential influence of transverse structural dimensions on material intensities and environmental indicators [62]. Therefore, to further verify the robustness of the results, a simple sensitivity analysis was conducted by adopting deck area (t/m²) as an alternative functional unit. This analysis was based on statistical estimates of lane numbers and deck widths for different bridge types, aiming to examine how alternative normalization approaches affect the internal trends of material intensities and environmental impacts, thereby providing a methodological basis for the subsequent discussion (detailed data are provided in the S1 File).The analysis indicates that, although absolute values of material intensities and environmental indicators differ under alternative functional units, the overall relative trends between materials and environmental impacts remain consistent. Therefore, using bridge length (t/km) as the primary functional unit for subsequent discussion effectively captures the material–environment trends and ensures the robustness and representativeness of the conclusions.

**Fig 6:**
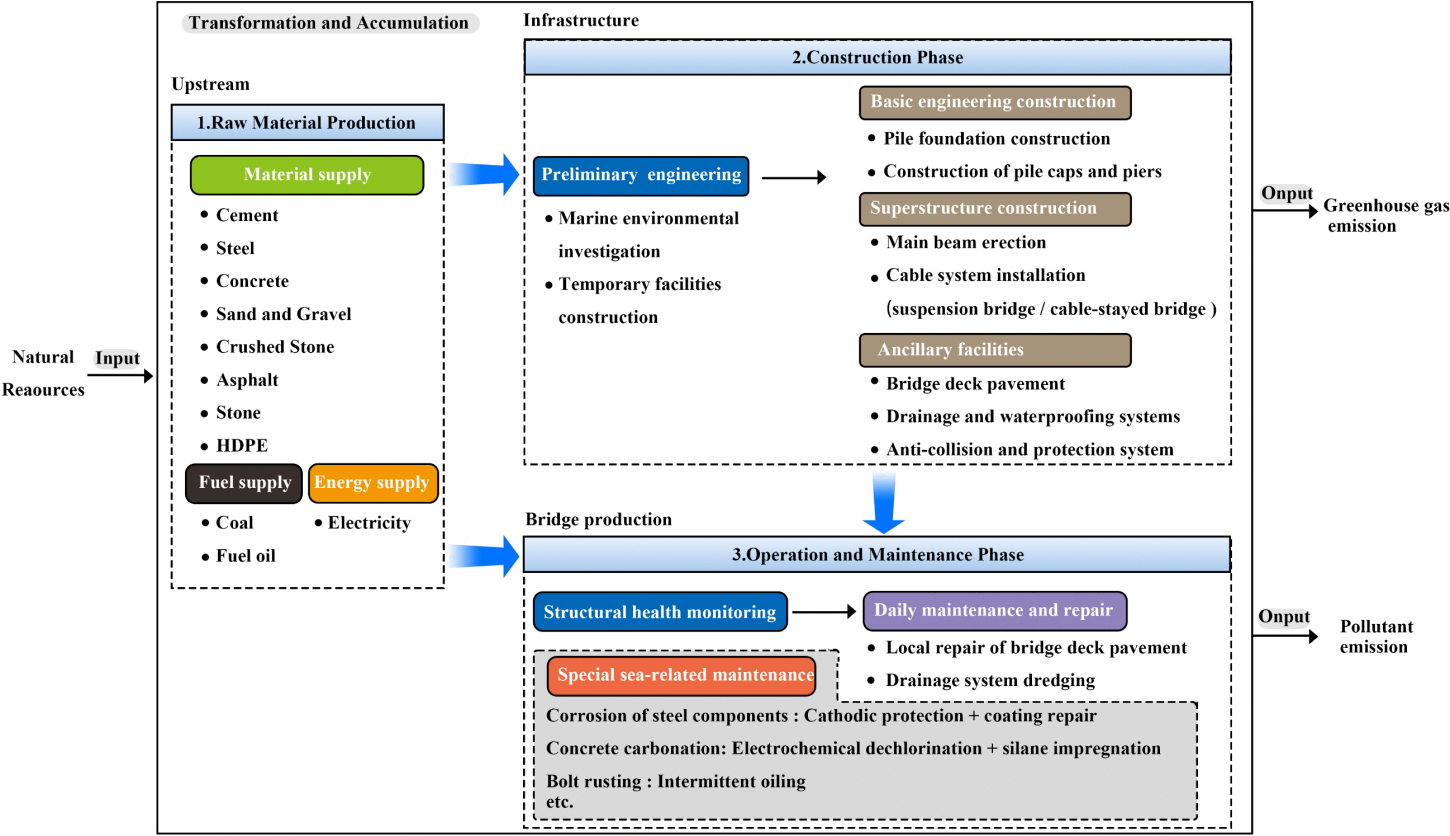
Boundary of the lifecycle system of coastal bridges. This diagram outlines the life cycle assessment for coastal bridge construction, covering production of materials, transportation, construction, and the ongoing operation and maintenance. It highlights energy demands, material accumulation, and environmental impacts.

Data collection is categorized into four types of bridge stock, and the material consumption per unit length of China’s coastal bridges is divided into six impact indicators: Global Warming Potential (GWP), Mineral Resource Scarcity, Fossil Resource Scarcity, Human Carcinogenic Toxicity, Freshwater Ecotoxicity, and Marine Ecotoxicity. Life cycle environmental impact assessment is conducted. As infrastructure products, coastal bridges have a lifecycle consisting of five main stages: from the production and processing of raw materials, to on-site construction, to the daily operation and maintenance of the bridges, and finally to the disposal and demolition phase. Given the high complexity and uncertainty associated with the recycling of materials and waste management during the disposal and demolition phase of coastal bridges, the system boundaries of this study primarily include the production and processing of raw materials, on-site construction, and the operation and maintenance stages of the bridges. The lifecycle process of coastal bridges is detailed in Fig 6.

#### 4.2.2. Life cycle inventory analysis based on SimaPro.

Based on the MS model, the quality of various material resources serves as the output data for the raw material production and processing phase. Combined with published literature, construction design documents, statistical yearbooks, and the Eco-invent database mentioned in Section 3.3, a lifecycle inventory system for China’s coastal bridge products has been established. The basis for this estimation is presented in Table 5.

**Table 5:** Life cycle inventory for Chinese coastal bridges per kilometer.

Bridge Type	Phase	Material/Energy Input	Estimated Value	Unit
Cable-Stayed Bridge	Raw Material Production	Cement	0.90	Mt/km
		Steel	1.01	Mt/km
		Asphalt	0.07	Mt/km
		Concrete	1.35	Mt/km
		Other (Bitumen and other materials)	2.61	Mt/km
		Electricity	3.32E + 08	kWh
	Construction Phase	Material Transport Machinery	915	p
		Construction Machinery	6,710	p
		Fuel	5.00E + 10	kWh
		Electricity	3.77E + 07	kWh
	Operation and Maintenance Phase	Facility Repair	6,739.84	m^2^
		Bridge Deck Repair	--	m^2^
		Spray Polyurea Elastomer	34,094	kg
		Electricity	--	kWh
Suspension Bridge	Raw Material Production	Cement	2.23	Mt/km
		Steel	0.91	Mt/km
		Asphalt	0.14	Mt/km
		Concrete	6.38	Mt/km
		Other (Bitumen and other materials)	9.09	Mt/km
		Electricity	2.91E + 07	kWh
	Construction Phase	Material Transport Machinery	243	p
		Construction Machinery	1,782	p
		Fuel	1.34E + 09	kWh
		Electricity	1.01E + 05	kWh
	Operation and Maintenance Phase	Facility Repair	2,517.85	m^2^
		Bridge Deck Repair	--	m^2^
		Spray Polyurea Elastomer	20,835	kg
		Electricity	--	kWh
Arch Bridge	Raw Material Production	Cement	0.18	Mt/km
		Steel	0.24	Mt/km
		Asphalt	0.01	Mt/km
		Concrete	0.24	Mt/km
		Other (Bitumen and other materials)	0.53	Mt/km
		Electricity	2.10E + 07	kWh
	Construction Phase	Material Transport Machinery	201	p
		Construction Machinery	1,474	p
		Fuel	1.11E + 09	kWh
		Electricity	8.39E + 04	kWh
	Operation and Maintenance Phase	Facility Repair	2,082.66	m^2^
		Bridge Deck Repair	--	m^2^
		Spray Polyurea Elastomer	16,505	kg
		Electricity	--	kWh
Beam Bridge	Raw Material Production	Cement	0.75	Mt/km
		Steel	0.83	Mt/km
		Asphalt	0.05	Mt/km
		Concrete	1.04	Mt/km
		Other (Bitumen and other materials)	2.02	Mt/km
		Electricity	2.68E + 07	kWh
	Construction Phase	Material Transport Machinery	171	p
		Construction Machinery	1,254	p
		Fuel	9.45E + 08	kWh
		Electricity	4,070,28	kWh
	Operation and Maintenance Phase	Facility Repair	1,771.82	m^2^
		Bridge Deck Repair	--	m^2^
		Spray Polyurea Elastomer	27,329	kg
		Electricity	--	kWh

Caption credit: ①The estimates for electricity and energy consumption in the table are based on the research results of scholars such as Liu MY et al. [63], Zhang LY et al. [64], Zhang YJ et al. [65], Hammervold et al. [66], Nuss et al [67] and include publicly available bridge construction data. The specific estimated values for electricity consumption and fuel for machinery used during the construction phase are provided in Table 2. ②For the operation and maintenance phase, the service life referred to in the study by Liu YY et al. [68] on the construction of a lifecycle carbon dioxide emission system for highways is used, with a design life of 100 years under ideal conditions. Based on this, the total electricity required for China’s coastal bridges is estimated to be 9.078776E9 kWh. The area of bridge deck maintenance for China’s coastal bridges, based on the research results of Huang W et al [69]., includes estimates for the use of materials such as concrete, asphalt, and cement, totaling approximately 3,825 ha.

#### 4.2.3. Phase-Specific contributions to environmental impacts.

**Fig 7:**
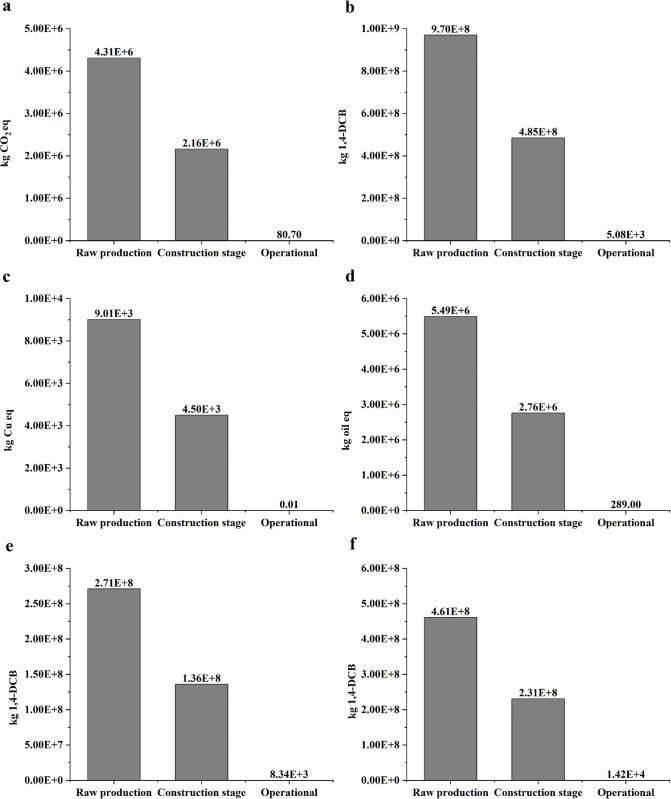
Phase-specific contributions to environmental impacts per km of bridge infrastructure. **(a)** Global warming potential (GWP) contributions by phase; **(b)** Human carcinogenic toxicity contributions by phase; **(c)** Mineral resource scarcity contributions by phase; **(d)** Fossil resource scarcity contributions by phase; **(e)** Freshwater ecotoxicity contributions by phase; **(f)** Marine ecotoxicity contributions by phase.

**Fig 8:**
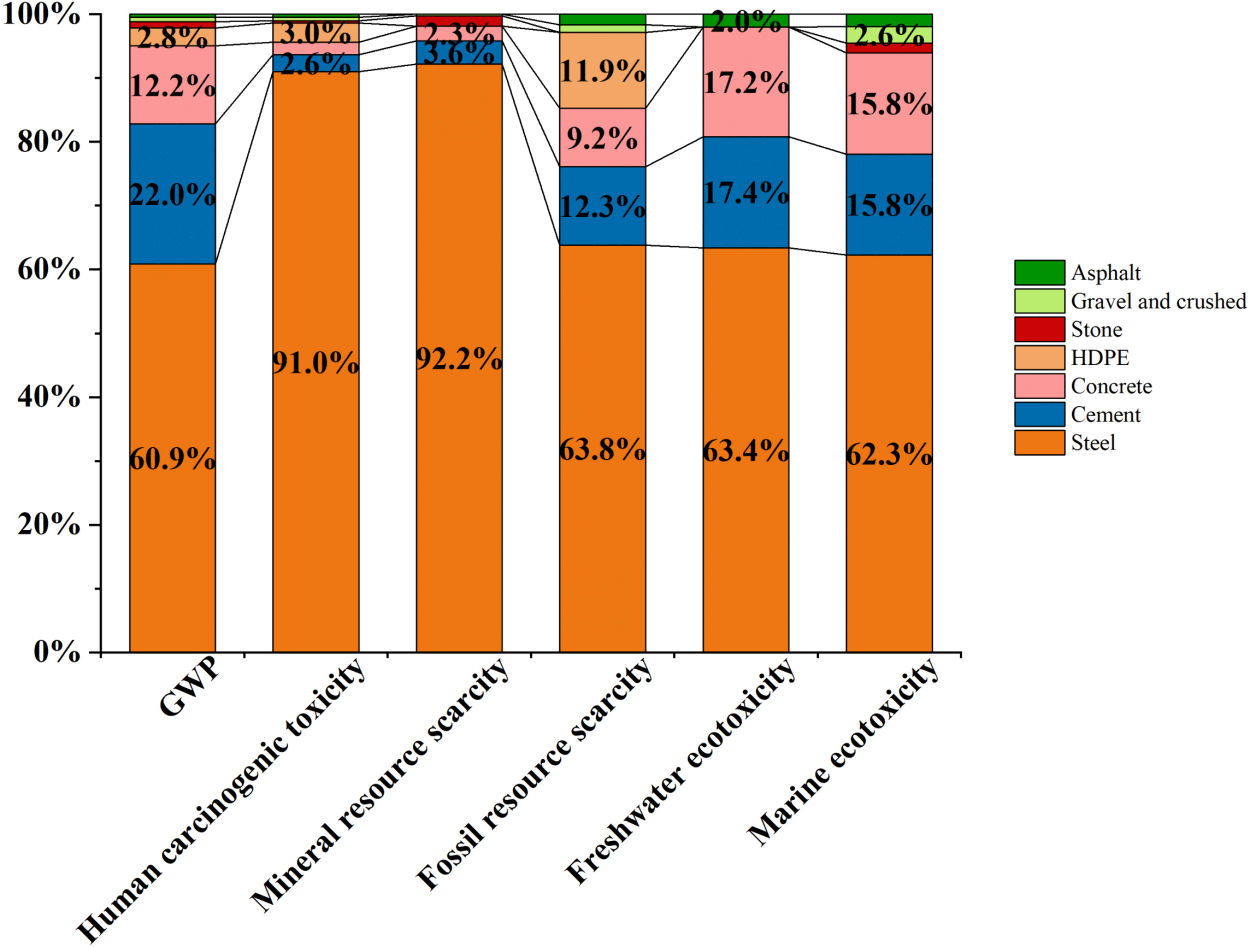
Contribution of raw material production to six environmental impact categories. (/km) Caption credit: less than 2% is not displayed.

Fig 7a-f and Fig 8 quantify the contributions of raw material production, construction, and operational phases and materials to six key environmental impact categories. Results are normalized per kilometer (km) of bridge length, revealing distinct patterns across impact categories.

**4.2.3.1 Global warming potential (GWP):** As shown in Fig 7a and Fig 8, greenhouse gas (GHG) emissions are predominantly attributed to raw material production, accounting for 66.6% (4.31 × 10^6^ kg CO₂ eq/km). Within this phase, steel smelting is the major contributor, responsible for 60.9% (2.62 × 10^6^ kg CO₂ eq/km), followed by cement clinker production at 22.0% (9.45 × 10^5^ kg CO₂ eq/km). The construction phase contributes 33.4% (2.16 × 10^6^ kg CO₂ eq/km), primarily due to diesel combustion from marine construction equipment (62.3%). The operational phase contributes a negligible share of 0.001% (80.70 kg CO₂ eq/km), indicating limited inclusion of long-term maintenance emissions.

**4.2.3.2 Human carcinogenic toxicity:** According to Fig 7b and Fig 8, material production accounts for 66.7% (9.70 × 10^8^ kg 1,4-DCB/km) of the total carcinogenic impact, stemming from three major sources: 1) Cement manufacturing: Cr⁶ ⁺ emissions from clinker grinding and kiln operations (2.6%, 2.54 × 10^7^ kg 1,4-DCB/km); 2) Steel industry: PAH emissions from coke oven gas and coal tar processing (91.0%, 8.83 × 10^8^ kg 1,4-DCB/km); 3) HDPE polymerization: Benzene and ethylene oxide releases (3.0%, 2.89 × 10^7^ kg 1,4-DCB/km). Construction activities contribute 33.3% (4.85 × 10^8^ kg 1,4-DCB/km), primarily due to VOC emissions from asphalt and particulates from welding fumes. Operational phase emissions are minimal, at 0.0004% (5.08 × 10^3^ kg 1,4-DCB/km).

**4.2.3.3 Mineral resource scarcity:** Fig 7c and Fig 8 illustrate that 66.7% (9.01 × 10^3^ kg Cu eq/km) of mineral depletion arises from raw material extraction. This is largely due to reinforcing steel production (92.2%, 8.30 × 10^3^ kg Cu eq/km), with additional contributions from cement and concrete (5.9%). The construction phase contributes 33.3% (4.50 × 10^3^ kg Cu eq/km), primarily from the use of temporary steel structures. The operational phase impact is negligible (0.00008%, 0.01 kg Cu eq/km).

**4.2.3.4 Fossil resource scarcity:** As presented in Fig 7d and Fig 8, fossil fuel depletion is driven by raw material production (66.5%, 5.49 × 10^6^ kg oil eq/km). Within this, steel-related coking coal use accounts for 63.8%, and petrochemical-based materials (cement, HDPE, concrete) contribute 33.4%. The construction phase adds 33.5% (2.76 × 10^6^ kg oil eq/km), mainly from steel-intensive equipment production (62.7%). The operational phase impact remains marginal at 0.004% (289 kg oil eq/km).

**4.2.3.5 Freshwater ecotoxicity:** Based on Fig 7e and Fig 8, freshwater toxicity is largely caused by material production (66.6%, 2.71 × 10^8^ kg 1,4-DCB/km), primarily from mining wastewater rich in heavy metals (63.4%). Construction activities contribute 33.4% (1.36 × 10^8^ kg 1,4-DCB/km), mostly due to sediment resuspension during pile driving (93.2%). The operational phase accounts for only 0.002% (8.34 × 10^3^ kg 1,4-DCB/km).

**4.2.3.6 Marine ecotoxicity:** As shown in Fig 7f and Fig 8, raw material production is responsible for 66.6% (4.61 × 10^8^ kg 1,4-DCB/km) of marine ecotoxicity, with steel manufacturing as the most critical stressor (62.3%, 2.87 × 10^8^ kg 1,4-DCB/km). Construction phase contributes 33.4% (2.31 × 10^8^ kg 1,4-DCB/km), driven by the use of zinc-rich epoxy paint (60–80% Zn) applied to metal coatings (62.3% of construction-phase marine toxicity). The operational phase impact is minimal (0.002%, 1.42 × 10^4^ kg 1,4-DCB/km), emphasizing the dominant role of upstream processes in generating marine toxic loads.

## 5. Discussion

### 5.1. Systemic drivers of lifecycle impacts

The systemic dominance is quantified by the material production phase, accounting for 66.6% of total impacts across all six categories (Fig 7), establishing it as the critical lifecycle bottleneck for China’s coastal bridges. This systemic reliance stems from carbon-intensive metallurgical processes, resource-depleting extraction practices, and region-specific industrial amplification effects, collectively entrenching environmental burdens in upstream supply chains.

#### 5.1.1. Material production as systemic bottleneck.

The life cycle environmental status of China’s coastal bridges presents a unique material production stage leading mode, dominating due to their special marine engineering properties and material technology system. This mode is consistent with global survey results of Sweden’s highway bridges, wherein the material phase accounts for 72–94% of the highway bridges total impact [70]. Cross sea bridges must contend with harsh environments such as high salt fog, strong corrosion, and deep-water foundations, resulting in sharply elevated material performance requirements. This highlights the universality of environmental costs in global bridge construction, especially in the energy-intensive production of steel and cement (Table 6).

**Table 6:** Percentage contributions of steel and cement to global warming potential and resource scarcity.

Impact Category	Steel Contribution	Cement Contribution
Global Warming Potential	60.9%	22.0%
Mineral Resource Scarcity	92.2%	3.6%
Fossil Resource Scarcity	63.8%	12.3%

Steel production accounts for 60.9% of global warming potential (GWP) and 63.8% of fossil resource scarcity (Table 5), reflecting China’s heavy reliance on blast furnace-basic oxygen furnace (BF-BOF) processes. BF-BOF accounts for 71.1% of China’s crude steel output, emitting 1.91 t CO_2_ eq per ton of steel [71], 15–20% higher than the global average (1.92 t CO_2_ eq/t steel) [70].

Cement production contributes 22.0% to the global warming potential (GWP) and the scarcity of fossil resources contributes 12.3% (Table 5), mainly due to its high-carbon process structure and resource dependence characteristics. According to “Research on the Carbon Neutral Path of China’s Cement Industry” [72], 95% of carbon emissions from cement production are concentrated in the clinker calcination process, of which process emissions (limestone decomposition) account for 60%, fuel Combustion (coal) accounts for 35%.

Coastal bridge construction intensifies environmental impacts. For instance, suspension bridges require 0.91 tons of steel and 2.24 tons of cement per kilometer (Table 3), amplifying the environmental footprint of steel and cement production. Steel’s overwhelming 92.2% contribution to mineral resource scarcity (Table 5) reflects its dependence on iron ore extraction (1.37 t ore/t steel, compared to the global average of 1.18 t ore/t steel) [73]. In contrast, cement’s minimal contribution (3.6%) underscores its reliance on limestone rather than scarce minerals.

#### 5.1.2. Hidden flows in operational systems.

The operational phase of coastal bridges demonstrates negligible direct environmental impacts across impact categories (<0.004%), compared to 12–15% of freshwater ecological toxicity in the European railway network caused by maintenance activities in the land transportation system [74]. This minimum operational and maintenance environmental contribution value reflects the particularity of a hundred-year design life that enhances corrosion resistance, such as galvanized steel coatings and HDPE protective layers [75–77].

However, this apparent low impact masks critical hidden flows that are systematically excluded from conventional LCAs [78]. In fact, these hidden flows are conceptually embedded in the design phase and are reflected in the life cycle inventory (LCI) of the raw material production and construction stages—through measures such as material redundancy, protective coatings, and anticipated maintenance—which implicitly account for the actual demands of the operational phase. As a result, the environmental burdens associated with these flows are largely shifted to the upstream stages, leading to an apparent contribution of the operational phase that is negligible in the ReCiPe 2016 Midpoint assessment (<0.004%). These hidden flows arise from three interrelated mechanisms: material overdesign to counteract corrosion, continuous leakage of toxic byproducts, and delayed maintenance burdens. All of these shift environmental impacts to upstream phases (material production and construction), accounting for more than 99% of the total impact across all six categories), while escaping operational-phase accounting. Within the static LCA framework and following the ReCiPe 2016 Midpoint method, the environmental burdens associated with these hidden mechanisms, such as additional material use and maintenance requirements as summarized in Table 7, effectively manifest in the upstream stages. Conceptually, this represents an internal redistribution within the system boundary. Without altering the overall balance of lifecycle impacts, the model maintains its closure and mass balance while highlighting the relative contribution of hidden flows to upstream environmental pressures. This perspective underscores that the negligible apparent impact of the operational phase does not indicate a lack of environmental significance, but rather a temporal and spatial displacement of burdens.

**Table 7:** Hidden operational flows in coastal bridges: Mechanistic drivers and quantified impacts.

Mechanisms	Element	Impact category	Value	Source
Material overdesign	Steel reinforcement	GWP	1.8 × 10^6^	ISO 9223–2012-classified corrosion rates (http://www.ecorr.org.cn/qita/new1/2020-05-18/177101.html) and Chinese engineering codes (GB/T 50476)
Continuous Leakage	Zinc leaching	Marine Ecotoxicity	2.3 × 10^8^ kg 1,4-DCB/km	ISO 9223 CX-classified corrosion rates (http://www.ecorr.org.cn/qita/new1/2020-05-18/177101.html)
Delayed Maintenance Burden	Asphalt PAHs	Human Carcinogenicity	2.1 × 10^5^ kg 1,4-DCB/km/cycle	NBS China (2022) (https://www.stats.gov.cn/english/)
	HDPE replacement	Freshwater Ecotoxicity	8.7 × 10^3^ kg 1,4-DCB/km	Plastics Europe (2023) (https://plasticseurope.org/knowledge-hub/plastics-the-fast-facts-2023/)

Chinese coastal bridges require 8–12% overdesign in steel (GB/T 50476) to offset 100-year corrosion losses (ISO 9223 CX: 0.12 mm/year), driving “invisible” carbon emissions during material production. This durability-driven overdesign contributes 1.8 × 10^6^ kg CO₂ eq/km (Table 6, 27.8% of material-phase GWP), embedding hidden carbon costs into bridge production while artificially minimizing operational carbon emissions.

Corrosion byproducts further drive hidden environmental flows: zinc-rich epoxy coatings release 2.3 × 10^8^ kg 1,4-DCB/km into marine ecosystems via tidal leaching(49.9% of material-phase marine ecosystems) [79]. Delayed maintenance activities – including 15-year asphalt recoating cycles (2.1 × 10^5^ kg 1,4-DCB/km/cycle) and HDPE replacements (8.7 × 10^3^ kg 1,4-DCB/km) – cumulatively emit carcinogens equivalent to 6.9% of construction-phase impacts (4.85 × 10^8^ kg 1,4-DCB/km). These design shifts environmental burdens from the operational phase to material production and construction (over 99% of total impacts).

Although effective in quantifying upstream impacts, the cradle-to-gate life-cycle assessment framework has limitations in capturing the degradation and maintenance dynamics of coastal bridges over time. Hidden flows were incorporated through static adjustments to the LCI or LCIA for quantitative representation. However, limited temporal data and predictive parameters constrain these estimates within a static framework, without capturing time-dependent factors such as degradation, maintenance, or service-life extension. Recent studies [80–82] further revealed that bridge structural behavior is scale-dependent and time-evolving, implying that lifecycle assessments should integrate time-dependent effects of structural degradation and hidden operational flows. To address this, dynamic LCA [83–85] can be integrated into infrastructure planning. Dynamic LCA accounts for time-varying factors such as material corrosion rates, maintenance intervals, and policy-driven technological shifts, enabling a more comprehensive assessment of hidden flows. By incorporating time-series data or probabilistic decay functions, dynamic LCA introduces four dynamic attributes—technological progress, variation in occupancy behavior, dynamic characteristic factors, and dynamic weighting factors—into a static LCA model. This allows the temporal evolution of processes to be simulated and environmental burdens to be distributed across the infrastructure’s entire service life, rather than concentrated in a single stage. A key distinction from static LCA lies in the use of time-varying input data. For example, protective coatings gradually corrode and leach, releasing heavy metals and microplastics into the marine environment; the associated burdens can be estimated using corrosion/leaching rates or empirical degradation functions and allocated across relevant lifecycle stages. Similarly, delayed maintenance can lead to structural stress accumulation and material fatigue, eventually triggering sudden repair activities that sharply increase resource and energy consumption; such burdens can be assessed using maintenance schedules, structural reliability models, or probabilistic repair functions. Compared with static frameworks, dynamic LCA more accurately captures the temporal evolution of hidden flows and the long-term impacts of material degradation and maintenance, making it particularly suitable for large, long-lived infrastructures, including sea-crossing bridges.

### 5.2. Material-intensive growth and lifecycle trade-offs in China’s marine infrastructure development

#### 5.2.1. Material-environment nexus in coastal bridge systems.

**Fig 9:**
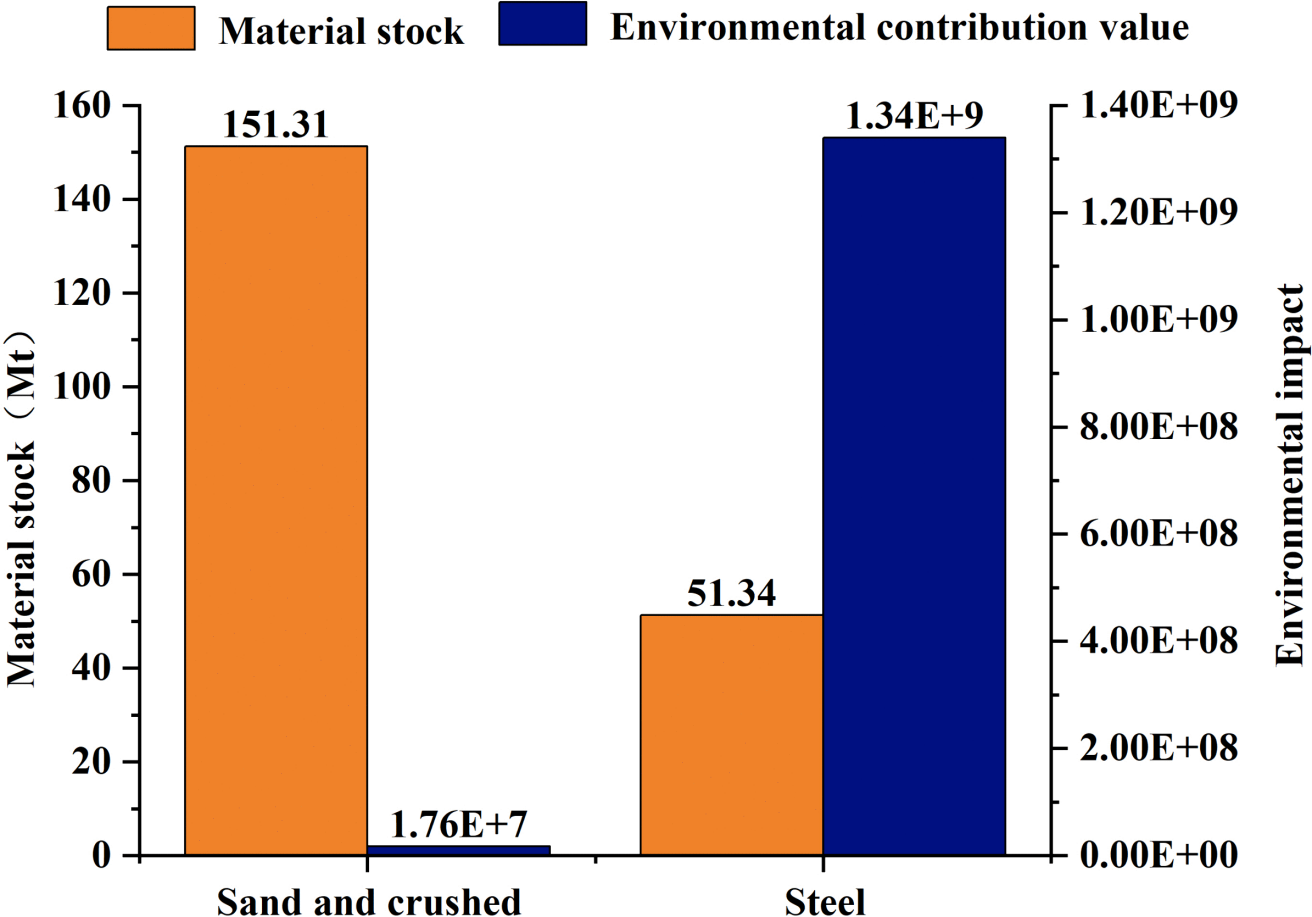
Comparison of material inventory and environmental impact value between sand and steel.

The material environment interaction in China’s coastal bridge system reveals a clear decoupling between material stock advantages and environmental impact intensity. Sand and crushed account for 40.97% (151.34 million tons, Fig 9) of the total material stock, with the smallest contribution to all six environmental indicators (<5%), while steel only accounts for 13.90% (51.34 million tons, Fig 9) of the material stock, representing 60.86% of the global warming potential (2.62 × 10^6^ kg CO_2_ eq/km), 91.03% of human carcinogenicity (8.83 × 10^8^ kg 1,4-DCB/km), and 92.21% of mineral scarcity impact (8.30 × 10^3^ kg Cu eq/km, Fig 8). The single environmental impact value is as high as 1.34 × 10^9^, which is several hundred times that of sand and crushed stone (Fig 9), attributed to highly correlated emissions of toxic heavy metals such as lead and cadmium, Zinc, copper, nickel, manganese [86–89].

It also significantly contributes to marine ecological toxicity (62.29%, 4.61 × 10^8^ kg 1,4-DCB/km), and the leaching of zinc and copper from anti-corrosion treatments (such as galvanized coatings) may synergistically interact with saline marine environments. amplifying the risk of bioaccumulation [90].This paradox stems from the fundamental difference between material service efficiency and structural demand hierarchy. According to the Sustainable Development Indicators report [91] released by the World Steel Association, compared to sand and gravel systems mainly used as low-energy structural fillers (such as bridge piers and roadbeds), the energy intensive metallurgical workflow of steel requires a higher energy intensity (21.27 gigajoules per ton of crude steel).

The structural type further amplifies this imbalance, as suspension bridges require 12.4 times more gravel (5.11 metric tons/kilometer) and 4.7 times more concrete (6.39 metric tons/kilometer) than cable-stayed bridge designs to meet anchoring system requirements [82,92], prioritizing mechanical stability over material efficiency. As demonstrated by Huang et al. [93], strengthening measures such as high-performance ferrocement laminates (HPFL) and bonded steel plates (BSP) can directly influence material durability and maintenance needs, thereby affecting lifecycle environmental burdens.

#### 5.2.2. Mitigation pathways for steel-driven environmental burdens and EoL Recycling Potential.

Across the six midpoint categories of ReCiPe 2016, steel is the dominant driver of environmental impacts in all categories (Fig 8; S1 File), accounting for approximately 60.9–92.2% of the total cross-category burden. On a per-ton basis, its impact intensity is estimated to be tens to hundreds of times greater than that of sand and gravel (Fig 9), reflecting its “low-mass, high-environmental-burden” characteristic, which arises from energy-, carbon-, and resource-intensive production processes [94]. Consequently, steel constitutes a critical bottleneck for the sustainable development of offshore bridge infrastructure, underscoring the need for integrated mitigation strategies through material innovation and structural optimization.

Reducing the steel-driven environmental burden requires a dual strategy of material substitution and structural innovation. Increasing the utilization rate of recycled steel can reduce the global warming potential (GWP) per kilometer of the bridge by 28–32%. Taking the data released by the China National Bureau of Statistics in 2022 as an example, the amount of waste steel recycling in the country is 120 million tons. For each ton of waste steel recycled, 1.5 tons of iron ore mining can be reduced, 86% of wastewater emissions can be reduced, 76% of waste slag generation can be reduced, 40% of energy consumption can be saved, and 1.6 tons of carbon dioxide emissions can be reduced. These savings primarily originate from avoided raw material extraction and reduced energy intensity in secondary steelmaking compared to primary production.

Building on this mechanism, a scenario analysis was conducted focusing on material recycling at the EoL stage to assess its potential in alleviating upstream environmental burdens. This analysis centers on steel as the primary environmental impact driver, while also incorporating cement—a high-emission material—to capture the synergistic mitigation effects of multi-material recycling and material innovation. The results indicate that increasing the recycling rate of steel to 10%, 20%, and 30% can reduce upstream global warming potential (GWP) by approximately 3%, 6%, and 9%, respectively, whereas improving cement recycling yields an additional 1%−3% reduction. These mitigation effects arise from the functional substitution of recycled materials for primary production processes, such as energy-intensive steel smelting and cement clinker calcination. Recycled cement is further reused in secondary concrete production, thereby reducing energy consumption and associated emissions during material manufacturing. In addition, the reduced demand for primary materials can indirectly alleviate environmental burdens during the construction and maintenance stages, including energy use for material transportation, on-site processing, and periodic bridge repair over the full-service life.

At the same time, the adoption of a cable-stayed–suspension hybrid design can reduce anchorage concrete demand by 12.4% per kilometer. As the world’s first double-layer hybrid bridge, the Third Yangtze River Bridge in Tongling (2020) demonstrates up to 80–90% reductions in concrete and steel use per unit span by distributing loads more efficiently, thereby minimizing the need for large anchorage blocks and tower foundations.

Addressing steel-induced environmental impacts requires not only material and design innovation but also systemic policy shifts that integrate lifecycle thinking across the planning, construction, and operational stages. Building on this and considering the potential of future end-of-life material recovery to offset upstream impacts, this study proposes five integrated pathways to support China’s carbon peaking and neutrality goals while enhancing the sustainability of coastal bridge infrastructure: 1) Expand the coverage of China’s national Emission Trading System (ETS) to progressively include the infrastructure sector. In accordance with the Interim Regulations on National Carbon Emission Trading and the Action Plan for Carbon Peaking in the Iron and Steel Industry (2022), carbon pricing mechanisms and fiscal incentives should be implemented to promote low-carbon cement and steel production through alternative fuels, hydrogen metallurgy, and carbon capture technologies; 2) Establish a comprehensive life-cycle management and recycling framework for marine infrastructure. Following the 14th Five-Year Plan for Circular Economy Development and the Steel Industry Adjustment and Upgrading Plan (2016–2020), increasing the share of recycled steel (e.g., ≈ 30%) and improving future recycling rates can enable closed-loop management across construction, operation, and regeneration phases, thereby mitigating overall environmental burdens; 3) Prioritize the development and application of modular prefabricated bridge systems, bio-based anti-corrosion materials, and intelligent protection technologies (e.g., hybrid designs reducing steel use by about 58%), in line with the Guidelines on Promoting the Construction of New Urban Infrastructure and Building Resilient Cities. These innovations can effectively reduce energy consumption and marine ecotoxicity during construction; 4) Incorporate a dynamic life-cycle assessment (LCA) framework to capture time-dependent processes such as corrosion, maintenance, and structural degradation, improving the accuracy of long-term environmental performance evaluation and supporting evidence-based infrastructure planning; 5) Strengthen international coordination under emerging carbon accounting and crediting frameworks such as the EU Carbon Border Adjustment Mechanism (CBAM). Enhancing supply chain transparency and developing internationally compatible LCA and carbon accounting systems will further reinforce the global competitiveness and sustainability of China’s coastal infrastructure sector.

## 6. Conclusions

This study provides the first integrated assessment of material stock (MS) and lifecycle environmental burdens associated with China’s coastal bridge infrastructure, using a bottom-up material flow analysis and ReCiPe 2016 midpoint life cycle assessment. The findings offer critical insights into the environmental trade-offs underpinning large-scale marine engineering projects.

First, China’s coastal bridge system contains an in-use material stock of 369.32 million tons, with cable-stayed bridges accounting for 63.9%. However, suspension bridges demonstrate the highest material intensity per kilometer (18.78 t/km), primarily due to anchorage systems requiring 12.4 × more crushed stone and 4.7 × more concrete than cable-stayed bridges. This highlights the need for span-specific structural and material optimization to balance performance with resource efficiency.

Second, lifecycle results show that raw material production dominates environmental burdens, contributing 66.6% of total impacts across six indicators. Steel production alone accounts for 60.9% of GWP and over 90% of mineral scarcity and human carcinogenicity. Despite constituting only 13.9% of the total MS, steel generates up to 380 × greater environmental intensity per unit mass than sand and gravel, establishing it as the primary environmental bottleneck.

Third, the operational phase, though seemingly negligible (<0.004%), conceals upstream-shifted hidden flows arising from material overdesign (8–12% steel redundancy), anticorrosive leaching (zinc), and delayed maintenance. These hidden flows account for 27.8% of lifecycle GWP (1.8 × 10^6^ kg CO₂ eq/km) and 49.9% of marine ecotoxicity (2.3 × 10^8^ kg 1,4-DCB/km), revealing systemic blind spots in conventional cradle-to-gate assessments.

To address these challenges, this study proposes five integrated strategies: expanding carbon markets to infrastructure, establishing ocean-specific LCA standards, promoting recycled materials and modular low-carbon design, adopting dynamic LCA frameworks, and aligning with global carbon governance mechanisms. These pathways offer actionable solutions for accelerating the low-carbon and climate-resilient transition of China’s coastal infrastructure.

## Supporting information

S1 File S1 Supplementary_All_Bridge_Points and PropertyInfo.xlsx. Spatial and attribute dataset of all cross-sea bridges analyzed in this study, including geographic coordinates (longitude and latitude), bridge names, bridge lengths, and structural types. S2 Supplementary_FigureData_Minimal.xlsx. Minimal datasets required to reproduce all quantitative figures presented in the manuscript. S3 Supplementary_Results_MFA_LCI_Impact Indicators.xlsx. Material stocks, material intensity, SimaPro LCI inputs, and life cycle environmental impact indicators by stage and by material. README.txt. Overview of the supplementary materials, including file structure, descriptions of each supplementary dataset, variables and units, data sources, and methodological notes.
(ZIP)
